# Overexpression of β-carboxysomes increases photosynthesis and growth in *Synechocystis* sp. PCC 6803

**DOI:** 10.1093/plphys/kiag261

**Published:** 2026-05-06

**Authors:** Jinlu Hu, Kuo Zhao, Yu Chen, Xingwu Ge, Jing Yang, Gregory F Dykes, Jian-Yu Shi, Lu-Ning Liu

**Affiliations:** School of Life Science and Technology, Northwestern Polytechnical University, Xi’an, Shaanxi 710072, China; Institute of Systems, Molecular and Integrative Biology, University of Liverpool, Liverpool L69 7ZB, United Kingdom; Institute of Systems, Molecular and Integrative Biology, University of Liverpool, Liverpool L69 7ZB, United Kingdom; Institute of Systems, Molecular and Integrative Biology, University of Liverpool, Liverpool L69 7ZB, United Kingdom; Institute of Systems, Molecular and Integrative Biology, University of Liverpool, Liverpool L69 7ZB, United Kingdom; Institute of Systems, Molecular and Integrative Biology, University of Liverpool, Liverpool L69 7ZB, United Kingdom; Materials Innovation Factory and Department of Chemistry, University of Liverpool, Liverpool L7 3NY, United Kingdom; Institute of Systems, Molecular and Integrative Biology, University of Liverpool, Liverpool L69 7ZB, United Kingdom; School of Life Science and Technology, Northwestern Polytechnical University, Xi’an, Shaanxi 710072, China; Institute of Systems, Molecular and Integrative Biology, University of Liverpool, Liverpool L69 7ZB, United Kingdom; MOE Key Laboratory of Evolution and Marine Biodiversity, State Key Laboratory of Marine Food Processing and Safety Control & College of Marine Life Sciences, Ocean University of China, Qingdao, China

## Abstract

Cyanobacteria are photoautotrophic cell factories capable of converting carbon dioxide into valuable chemicals. The rate-limiting step in photosynthesis is the enzymatic carbon fixation catalyzed by Rubisco. The carboxysome is a protein-based organelle that plays a central role in cyanobacterial carbon fixation. It consists of thousands of subunits, including hexameric and pentameric proteins, which form a shell to encapsulate the enzymes Rubisco and carbonic anhydrase. Here, we enhanced β-carboxysome biogenesis in *Synechocystis* sp. PCC 6803 by overexpressing a full or partial set of endogenous β-carboxysome components. Overexpression of carboxysome proteins altered protein stoichiometry, morphology, and cellular abundance of β-carboxysomes, as well as improved photosynthetic activities and cell growth. These findings indicate that targeted carboxysome overexpression enhances photosynthetic efficiency and growth of cyanobacteria under favorable conditions. This study provides a framework for engineering cyanobacterial chassis cells as microbial cell factories and carboxysome-based CO_2_-concentrating mechanisms in heterologous hosts, including crop plants, to improve photosynthetic productivity for sustainable bioproduction.

## Introduction

The escalating global energy demand, coupled with heavy reliance on finite fossil fuels, has led to massive emissions of greenhouse gases, such as CO_2_, posing a severe threat to the environment (Wang and Azam 2024). Cyanobacteria, as evolutionary ancestors of algal and plant chloroplasts, offer a compelling avenue for carbon sequestration and bioresource production (Hu et al. 2023a). However, the average yields are often low compared to heterotrophic microbes, partly due to slower growth and lower biomass accumulation, which represent major bottlenecks for their commercial viability (Liu et al. 2024).

Photosynthesis is a vital biological process that drives energy conversion and underpins global carbon cycling, forming the foundation of virtually all life on Earth (Hohmann-Marriott and Blankenship 2011; Liu 2016; Mullineaux and Liu 2020). The 3 commonly acknowledged strategies to improve photosynthesis include improving light capture, enhancing photosynthetic electron transport, and optimizing the CO_2_-fixing enzyme Rubisco, along with relevant enzymes (Hu et al. 2023a). Rubisco suffers from severe catalytic inefficiency and competitive oxygenation, representing the rate-limiting step in photosynthesis (de Pins et al. 2025). To suppress the oxygenase reaction and enhance the carboxylation of Rubisco, distinct phototrophic organisms, including cyanobacteria, algae, C4 plants, and crassulacean acid metabolism (CAM) plants, have evolved various CO_2_-concentrating mechanisms (CCMs) to accumulate CO_2_ around Rubisco (Keeley and Rundel 2003; Fei et al. 2022; Sarkar et al. 2024; Sun et al. 2025).

The carboxysome serves as the core component of CCM in cyanobacteria and some proteobacteria and is classified into α-carboxysome and β-carboxysomes based on differences in their composition and assembly principles (Liu 2022; Nguyen et al. 2025b). In β-carboxysomes, cargo enzymes, including Rubisco (comprising large and small subunits RbcL and RbcS) and carbonic anhydrase (CA; also known as CcaA), are densely packed with the assistance of internal scaffolding proteins to form the enzyme core, which is encapsulated by a proteinaceous shell (Sun et al. 2025). The β-carboxysome shell is composed of a series of proteins encoded by the *ccm* operon, including the hexameric CcmK paralogs (CcmK1 to 4), that predominantly form shell facets (Kerfeld et al. 2005), the pentameric CcmL proteins that occupy the vertices of the polyhedron (Tanaka et al. 2008), and the trimeric proteins CcmO and CcmP (Cai et al. 2013; Larsson et al. 2017). The scaffolding proteins CcmM and CcmN have been proposed to act as adaptors that crosslink the enzymatic core to shell facets (Wang et al. 2019; Zang et al. 2021). CcmM exists as 2 isoforms, both of which are essential for β-carboxysome assembly. The full-length form contains a CA-like domain followed by 3 to 5 Rubisco small subunit-like domains connected by flexible linkers, while the shorter form lacks the N-terminal CA-like domain (Long et al. 2010). The short-form CcmM aggregates Rubisco molecules and induces phase separation into a liquid-like matrix (Wang et al. 2019), whereas the full-length CcmM interacts with the N-terminal region of CcmN to facilitate the recruitment of shell proteins and the assembly of intact β-carboxysome (Kinney et al. 2012; Sun et al. 2021). Recent studies have revealed that CcmS is required for the coordinated assembly of carboxysomes through its interaction with the CcmK1 C-terminus exposed at the outer surface of the shell (Chen et al. 2023b; Cheng et al. 2024). Moreover, auxiliary factors Raf1 and RbcX are involved in carboxysome formation and regulation (Huang et al. 2018; Huang et al. 2020).

Enhancing photosynthetic carbon fixation by modifying the expression of key genes in the relevant pathways holds great promise for synthetic biology aimed at increasing biomass production. In recent years, numerous mutant strains have been constructed to overexpress endogenous or exogenous genes encoding key enzymes involved in carbon fixation, including Rubisco and Rubisco activase, with the aim of identifying variants that exhibit enhanced growth and increased biomass accumulation (Liang and Lindblad 2017; Wei et al. 2017). Given the essential role of carboxysomes in photosynthetic carbon fixation, enhancing carboxysome biosynthesis in cells offers a promising strategy for supercharging photosynthesis and cell growth.

In this study, we generated constructs that overexpress endogenous carboxysome proteins in the model cyanobacterium *Synechocystis* sp. PCC 6803 (Syn6803), and systematically characterized the morphology and number of carboxysomes in bacteria, as well as the changes in cell growth and photosynthetic capacity. Our results reveal that overexpression of carboxysome proteins resulted in not only an increase in carboxysome number but also improved photosynthesis and growth in elevated CO_2_ and optimized medium. Our findings pave the way for engineering cyanobacterial chassis cells as microbial cell factories and constructing carboxysome-based structures in non-native organisms (eg crop plants) for biotechnological applications.

## Results and discussion

### Generation and characterization of Syn6803 mutants overexpressing genes involved in β-carboxysome assembly

**Figure 1:**
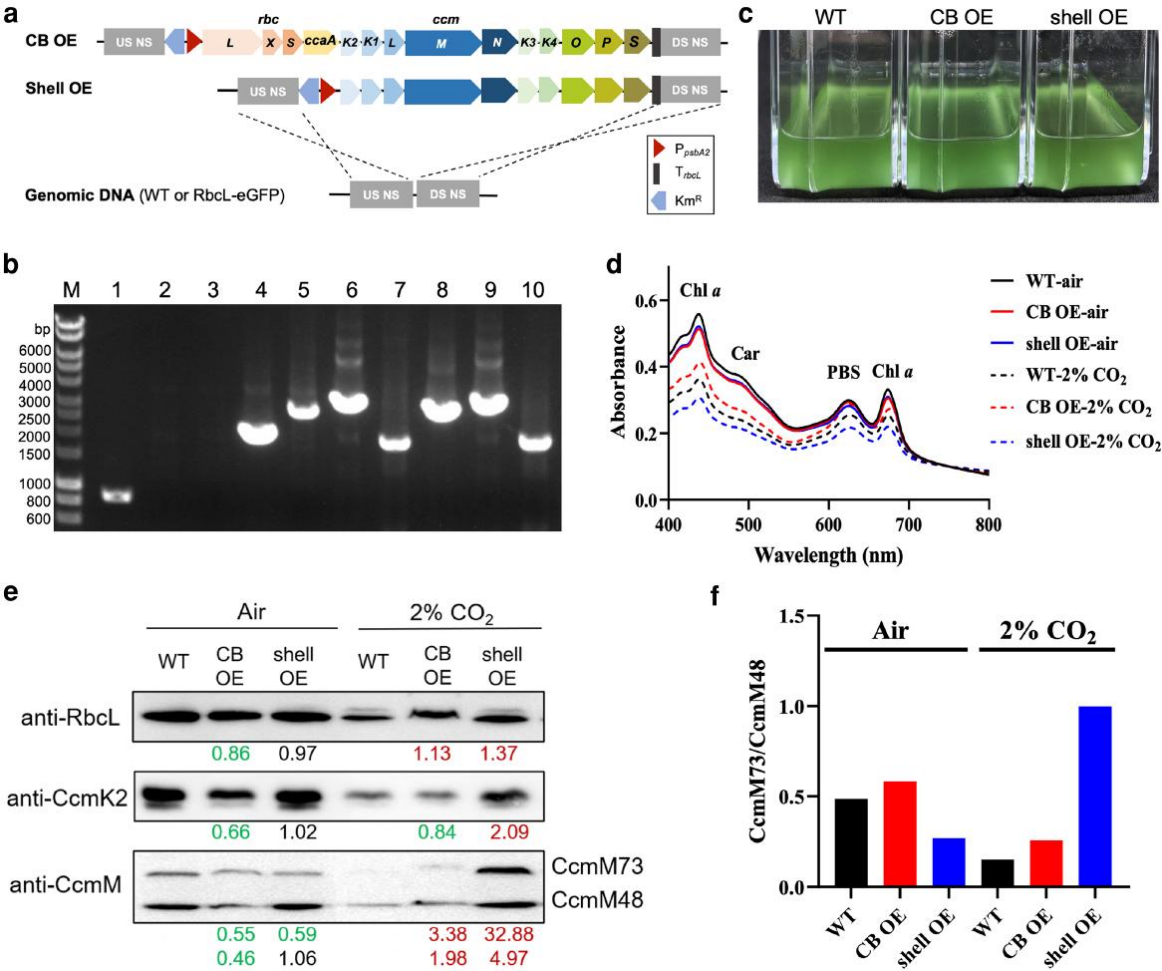
Construction and characterization of the Syn6803 CB OE and shell OE mutants. a) A graphical depiction of the genetic overexpression of endogenous β-carboxysome genetic components within the *slr0168* chromosomal neutral site (NS) by double homologous recombination. The full (CB OE) or partial (shell OE) set of β-carboxysome components were expressed with the *psbA2* promoter and the *rbcL* terminator. US, upstream; DS, downstream. b) PCR verification of full segregation of CB OE and shell OE mutants. The template of Lane 1 is the WT genome. The templates of Lanes 2 and 4 to 7 are the CB OE genome. The templates of Lanes 3 and 8 to 10 are the shell OE genome. The screened fragment of *slr0168* in the WT is 847 bp. c) Culture samples after 3 days of cultivation. d) Absorption spectra of whole cells of WT, CB OE, and shell OE strains in normal and 2% CO_2_ conditions. The absorbance spectra were normalized at 750 nm. Specific pigments were labeled above the peaks. Chl *a*, chlorophyll *a*; Car, carotenoids; PBS, phycobilisomes. e) Immunoblot of soluble protein extracts from WT and mutant cells. Each lane was loaded with 40 *μ*g of total protein. The numbers beneath the protein bands indicate the ratio of protein signal in the mutant strain relative to the WT level under identical culture conditions. Green values signify proteins downregulated by more than 10%, while red values denote proteins upregulated by more than 10%. f) Ratios of CcmM73/CcmM48.

Previous studies have highlighted the difficulty of substantially increasing Rubisco content in cyanobacteria and their growth rate through genetic engineering (Liang and Lindblad 2016, 2017). In this work, two Syn6803 mutants were generated using distinct genetic strategies to promote endogenous β-carboxysome expression (Fig. 1a). One mutant (designated CB OE) was generated by inserting 14 carboxysome genes encoding cargo, shell, and scaffolding components into the *slr0168* chromosomal neutral site under the *psbA2* promoter, in addition to native carboxysome expression. The other mutant (shell OE) overexpressed 10 genes encoding only shell and scaffolding proteins. Each plasmid was transformed into both the wild type (WT) and RbcL-GFP mutant cells to obtain homozygous mutant strains (Fig. 1b). Whole-cell absorption spectra of WT and mutants under ambient air conditions showed nearly identical chlorophyll *a*, carotenoid, and phycobilisome peaks, indicating that pigment composition and culture coloration were essentially unchanged (Fig. 1c and d). In contrast, when cultivated under 2% CO_2_, the CB OE mutant displayed a slight increase in the absorption peaks associated with these major photosynthetic pigments compared to the WT (Fig. 1d).

We assessed the expression of RbcL, CcmK2, and CcmM using immunoblot analysis (Fig. 1e). Under ambient air conditions, the levels of these carboxysome proteins in both mutants, particularly in the CB OE mutant, were reduced compared to those in the WT. Consistently, Rubisco overexpression in cyanobacteria and plants is often accompanied by a decline in Rubisco content and activation (Liang and Lindblad 2016; Suganami et al. 2020). These results suggest that native Rubisco enzymes have already been tuned close to their physiological optimum, despite their inherent catalytic limitations (Prayogo et al. 2020). In contrast, under 2% CO_2_, the abundance of carboxysome proteins notably increased compared to those in the WT, except for CcmK2 in the CB OE mutant. CO_2_ availability is a key regulatory factor promoting the biogenesis of β-carboxysomes (Long et al. 2021); higher CO_2_ reduced the Rubisco level and resulted in the formation of smaller carboxysomes in *Synechococcus elongatus* PCC 7942 (Sun et al. 2019). Consistently, in many plant species, Rubisco abundance generally declines under elevated CO_2_ conditions (Moore et al. 1998). Our results suggest that heterologous expression of synthetic carboxysomes effectively offsets the inhibitory influence of high CO_2_ on native carboxysome biosynthesis.

Carboxysome architecture is fundamental to its physical mechanics (Faulkner et al. 2017) and is dynamically regulated by environmental changes (Sun et al. 2019). CcmM plays an essential role in Rubisco nucleation (Wang et al. 2019), and the stoichiometric composition of CcmM isoforms is crucial for mediating β-carboxysome assembly and organization (Long et al. 2010; Sun et al. 2025). In Syn6803, CcmM exhibits 2 isoforms, CcmM73 and CcmM48 (Chen et al. 2023b). The CcmM73:CcmM48 ratio declined with increasing CO_2_ concentration and was modulated by the overexpression of carboxysomes or shell components (Fig. 1f). In a similar manner, carboxysomes from WT *Synechococcus* 7942 cells grown in air contained slightly higher levels of CcaA and CcmM58 but lower amounts of CcmM35 than those from cells cultivated under 2% CO_2_ (Long et al. 2011). Consistently, under high CO_2_ conditions, a lower CcmM58:CcmM35 ratio was determined for *Synechococcus* 7942, which coincided with a reduction in carboxysome number (Sun et al. 2019).

**Figure 2:**
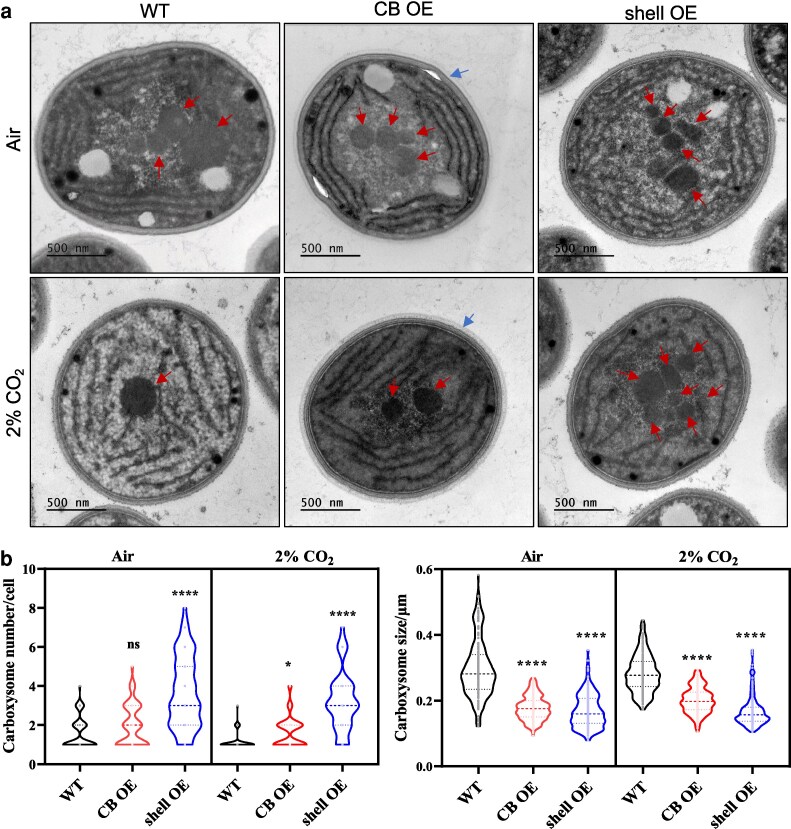
Organization and distribution of carboxysomes in the Syn6803 CB OE and shell OE mutants. a) Thin-section images of WT and mutant cells. Red arrows indicate the carboxysomes in the WT and mutant cells. Blue arrows likely indicate the enhanced production of S-layers or polysaccharides. b) Statistical characterization of carboxysome numbers per cell (n > 40, 1-way ANOVA followed by Dunnett's test) and sizes per carboxysome (n > 60, 1-way ANOVA followed by Dunnett's test) of the electron microscopy images. ns = no significance, *P* > 0.05; *0.01 < *P* < 0.05; ****P < 0.0001. The small, aligned dots spread across the violin plot represent the raw data points. The center dashed line represents the median, and the upper and lower dashed lines represent the quartiles (the 25th and 75th percentiles).

We questioned whether overexpressing carboxysome proteins and modified protein stoichiometry could alter carboxysome formation and structure in Syn6803. To address this, thin-section transmission electron microscopy (TEM) was employed to determine the carboxysome number and morphology in the CB OE and shell OE mutants grown under both ambient air and 2% CO_2_ (Fig. 2a). Under both growth conditions, the WT and CB OE cells possessed carboxysomes with a comparable polyhedral morphology; in contrast, the carboxysomes in shell OE cells displayed a slightly higher variability in morphology. Moreover, under ambient air, the shell OE mutant showed a notable increase in carboxysome abundance compared to the WT, whereas under 2% CO_2_, both mutants exhibited increased numbers of carboxysomes relative to the WT (Fig. 2b). Quantitative analysis indicated that, under air, the diameter of carboxysomes in CB OE (177.8 ± 2.3 nm, n = 88) and shell OE (168.8 ± 3.8 nm, n = 61) mutants was smaller than that of WT carboxysomes (296.5 ± 6.2 nm, n = 65) (Fig. 2b). A similar pattern was observed under 2% CO_2_, where WT carboxysomes averaged 283.1 ± 3.8 nm (n = 77) in diameter, compared to 201.3 ± 2.9 nm (n = 63) and 168.1 ± 3.4 nm (n = 64) in CB OE and shell OE cells, respectively (Fig. 2b). These results suggest that (i) CB OE and shell OE represent distinct stoichiometric perturbations, (ii) both mutants produce more but smaller carboxysomes relative to WT, and (iii) shell OE exhibits the strongest size reduction and highest number increase, suggesting that the altered stoichiometry of carboxysome proteins may hasten cargo envelopment, leading to the formation of more, but smaller, organelles (Sun et al. 2019; Sarkar et al. 2024; Trettel et al. 2025). Whether smaller carboxysomes confer a competitive advantage in substrate permeation and turnover merits further investigation.

**Figure 3:**
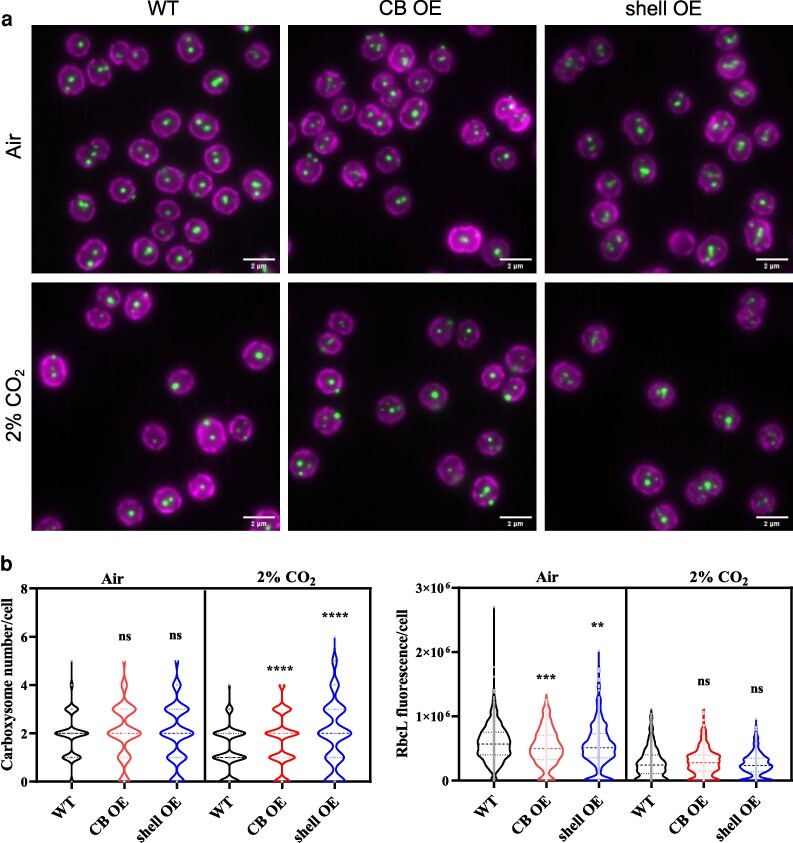
Organization of carboxysomes in the Syn6803 CB OE and shell OE mutants. a) Live-cell super-resolution fluorescence microscopy images of WT and mutants. Green signals represent RbcL labeled with GFP, indicating the carboxysome structures in the WT and mutant cells. Magenta signals represent the chlorophyll autofluorescence from thylakoid membranes. b) Quantification analysis of carboxysome numbers and fluorescence intensity per cell (n > 230, 1-way ANOVA followed by Dunnett's test) of the fluorescence images. ns = no significance, *P* > 0.05; **0.001 < *P* < 0.01; ***0.0001 < *P* < 0.001; ****P < 0.0001. The small, scattered dots spread across the violin plot represent the raw data points. The center dashed line represents the median, and the upper and lower dashed lines represent the quartiles (the 25th and 75th percentiles).

Native RbcL was labeled with GFP for in vivo visualization of carboxysomes under fluorescence microscopy (Sun et al. 2016; Sun et al. 2019; Huang et al. 2020; Sun et al. 2020). Live-cell imaging showed that most of the carboxysomes were located in the cytoplasm of round-shaped Syn6803 cells, whereas some were located nearby the thylakoid membranes (Fig. 3a), which is in agreement with previous TEM observations (McKay et al. 1993; Agarwal et al. 2009). Under ambient air, a slight increase in the number of carboxysomes per cell was observed in the CB OE and shell OE mutants, although not statistically significant, compared to that in the WT (in a range of 1 to 4 carboxysomes per cell) (Fig. 3b). It is speculated that a subset of carboxysomes in shell OE mutant cells may lack assembled Rubisco. In contrast, both mutants exhibited a remarkable increase in carboxysome number under 2% CO_2_ compared to the WT (Fig. 3b), consistent with our TEM results (Fig. 2b). Furthermore, the Rubisco content per cell as indicated by whole-cell RbcL-GFP fluorescence intensity was reduced in both mutants under air conditions (Fig. 3b), and no significant differences were observed under 2% CO_2_. Under ambient air, immunoblot analysis showed that RbcL abundance in the 2 mutants decreased to 86% and 97% of WT levels (Fig. 1e). Consistently, the Rubisco content per cell as indicated by RbcL-GFP fluorescence intensity was reduced to 88% and 92% of WT in the respective mutants (Fig. 3b). The close agreement between immunoblot and fluorescence measurements confirms that GFP tagging does not markedly affect RbcL expression, in line with previous observations (Sun et al. 2019).

Overall, our results indicate that overexpression of carboxysome proteins altered carboxysome number, morphology, and potential compositional organization in Syn6803, especially under 2% CO_2_ conditions.

### Growth and physiological properties of carboxysome-overexpressing strains

**Figure 4:**
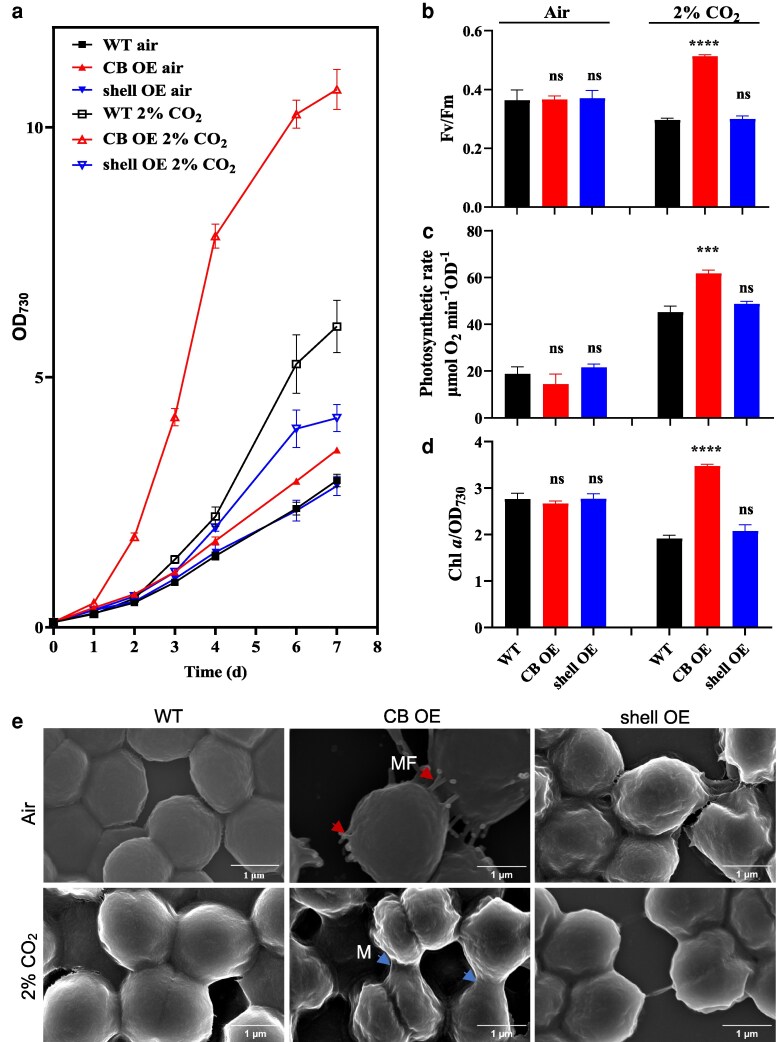
Comparison of growth and physiological parameters of CB OE and shell OE mutants under air or the presence of 2% CO_2_. a) Growth curve of all cyanobacterial strains; b) Fv/Fm; c) photosynthetic rate/OD_730_; d) chlorophyll *a*/OD_730_. For all growth curve experiments, 30 mL cultures adjusted to OD_730_ = 0.1 were grown in filter flasks. Data points with error bars represent the mean of n = 3 biological replicates ± Sd. ns = no significance, *P* > 0.05; ***0.0001 < *P* < 0.001; ****P < 0.0001. e) SEM of the Syn6803 WT, CB OE, and shell OE mutant cells. Red arrows indicate the mucus fibers (MFs) in the CB OE mutant cells. Blue arrows indicate the mucilaginous sheath (M).

We reasoned that overexpression of carboxysome proteins could enhance cell growth. To address this, we performed a comprehensive analysis to elucidate the effects of carboxysome protein overexpression on cell growth and physiology. Under air conditions, the growth rate of the CB OE and shell OE mutant strains remained unchanged compared to that of the WT (Fig. 4a). The photosynthetic parameters, including the maximum photochemical quantum yield of PSII (Fv/Fm), oxygen evolution rate, and pigment content, showed no significant differences among the strains (Fig. 4b to d; Fig. S1a).

Under 2% CO_2_, however, CB OE cells showed a substantially greater growth rate, reaching 1.8-fold the optical density of WT (Fig. 4a). This mutant also exhibited pronounced increases in Fv/Fm and oxygen evolution by 73% and 37%, respectively (Fig. 4b and c), and accumulated approximately 81% more chlorophyll *a* after 3 d of cultivation, whereas carotenoid content remained unchanged (Fig. 4d; Fig. S1a). These results indicate that enhanced carbon fixation can directly contribute to improved photosynthetic light reactions (Liang and Lindblad 2017). In contrast, the shell OE mutant showed growth and photosynthetic characteristics comparable to those of the WT. The observed variation may be attributed to the divergent stoichiometries of carboxysome components in the 2 mutant strains. These findings suggest that a balanced stoichiometry of carboxysome components is essential for specific carboxysome assembly and function (Sun et al. 2019; Liu et al. 2021; Sun et al. 2025).

An external electron-dense layer (S-layer-/sheath-like) was observed surrounding the CB OE cells by TEM, particularly under 2% CO_2_ (Fig. 2a). Consequently, we employed scanning electron microscopy (SEM) to investigate the effects of carboxysome protein overexpression on the cell surface appearance in comparison with the WT (Fig. 4e). Under ambient air, relative to the WT and shell OE strains, the CB OE mutant exhibited abundant mucus fibers on the surface of newly divided cells. Under 2% CO_2_, thick, connection-like mucilaginous sheaths were observed between daughter cells of the CB OE mutant. Furthermore, we found that the total carbohydrate contents did not differ significantly among the 3 strains under either condition (Fig. S1b). Therefore, the thick mucilaginous sheath observed in CB OE cells may reflect altered allocation and/or architecture of extracellular polymeric material (including exopolysaccharides (EPS)), potentially alongside increased S-layer protein abundance. The emergence of the specific structures around CB OE cells, especially under 2% CO_2_, further suggests a reallocation of fixed carbon toward extracellular polymer production and altered carbon flux, a phenomenon previously observed in cyanobacteria (Agarwal et al. 2018; Xiao et al. 2020; Eungrasamee et al. 2022).

### Effects of optimized conditions on the growth of CB OE and shell OE mutants

**Figure 5:**
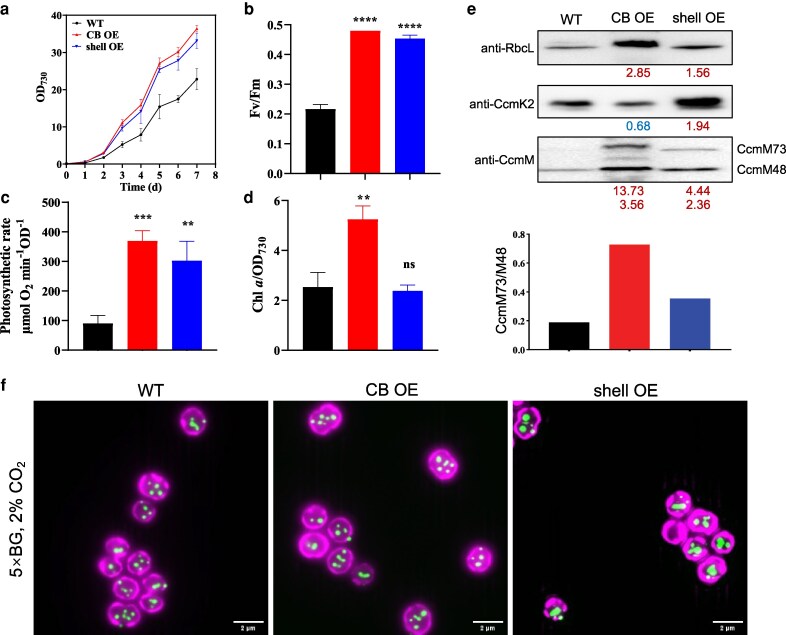
Effect of the medium on the growth of CB OE and shell OE mutants in the presence of 2% CO_2_. All strains were grown in 5× BG medium, with light intensity gradually increased from 50 to 100 *μ*mol photons m^−2^ s^−1^ (Day 1) and eventually set to 500 *μ*mol photons m^−2^ s^−1^ (Day 3). a) Growth curve of all cyanobacterial strains; b) Fv/Fm; c) photosynthetic rate/OD_730_; d) chlorophyll *a*/OD_730_; ns = no significance, *P* > 0.05; **0.001 < *P* < 0.01; ***0.0001 < *P* < 0.001; ****P < 0.0001. Data points with error bars represent the mean of n = 3 biological replicates ± Sd. e) Immunoblot of soluble protein extracts from WT and mutant cells after 7 d of cultivation. Each lane was loaded with 40 *μ*g of total protein. Ratios of CcmM73/CcmM48 are displayed at the bottom. f) Live-cell fluorescence microscopy images. Green signals represent RbcL labeled with GFP, indicating the carboxysome structures and subcellular organization within the WT and mutant cells. Magenta signals represent chlorophyll autofluorescence from thylakoid membranes.

Under high CO_2_, the CB OE mutant exhibited a 1.8-fold increase in growth compared to WT after 7 d of cultivation (Fig. 4a). To investigate whether this growth advantage could be further enhanced by alleviating common constraints in high-carbon conditions, specifically nutrient availability and light intensity, we evaluated cell growth under more favorable conditions, including nutrient-enriched medium, elevated CO_2_, and optimized illumination (Włodarczyk et al. 2020). For this purpose, CB OE and shell OE mutants were cultured in 5× concentrated BG-11 medium (5× BG) supplemented with 2% CO_2_ under continually optimized light to assess the impact of these conditions. Under these conditions, all cultures grew faster than in normal BG-11 medium (Fig. 5a). Specifically, both mutants exhibited substantially stimulated growth, reaching OD_730_ values of 36.4 and 33.2 after 7 d (Fig. 5a). Both mutants also displayed enhanced photosynthetic performance, as reflected by elevated Fv/Fm and oxygen evolution rates compared to WT levels (Fig. 5b and c). The increase in chlorophyll *a* content remained unique to the CB OE mutant, whereas carotenoid levels were unchanged in both mutants (Fig. 5d; Fig. S1a). Similarly, the total carbohydrate contents showed no substantial differences among the strains grown under 5× BG supplemented with 2% CO_2_ (Fig. S1b). Interestingly, under these optimized conditions, the CB OE mutant still displayed a greater growth advantage, reaching 1.6-fold the WT level (Fig. 5a). This finding suggests that its superior growth phenotype is most strongly expressed under high CO_2_ and does not increase proportionally as other environmental constraints are progressively relieved.

Immunoblot analysis revealed that the abundance of RbcL, CcmK2, and CcmM proteins increased substantially in 5× BG under 2% CO_2_ conditions, except for CcmK2 in the CB OE mutant (Fig. 5e). By providing more nutrients in addition to 2% CO_2_, the RbcL abundance in CB OE increased from 1.13- to 2.85- fold of that in WT. Additionally, remarkable alterations were observed in both the abundance and ratio of the 2 CcmM isoforms in the 2 mutants under nutrient-rich conditions. Live-cell fluorescence imaging revealed that the number of carboxysomes in all the strains substantially increased in 5× BG medium, possibly supporting their rapid growth under these conditions (Fig. 5f).

**Figure 6:**
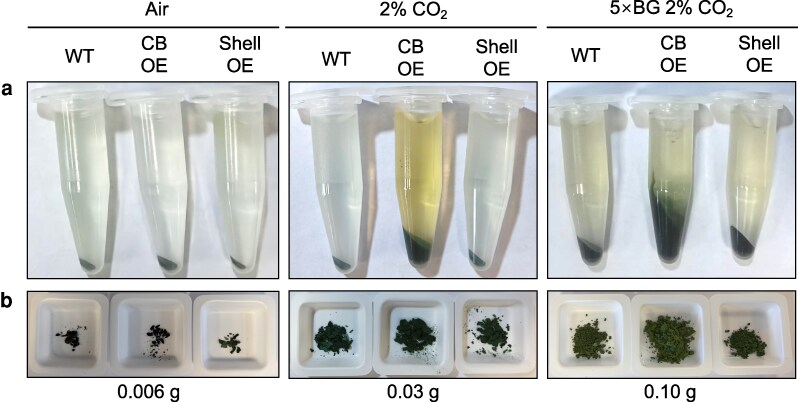
Comparison of growth performance and biomass accumulation under different CO_2_ and media conditions. a) Comparison of the cell pellet size from 1.5 mL of each culture harvested after 7 d of cultivation. For all growth curve experiments, 30 mL cultures adjusted to OD_730_ = 0.1 were grown in filter flasks. b) 0.006, 0.03, and 0.1 g dried powder samples under air, 2% CO_2_, and 2% CO_2_ in 5× BG medium.

In addition to elevated growth, we also found that the CB OE mutant displayed poor sedimentation during culture handling and centrifugation, especially under high CO_2_ and/or 5× BG conditions. After centrifugation, the CB OE cell pellet was visibly larger than those of the WT or shell OE strains (Fig. 6a) and the freeze-dried powder of CB OE cells formed loose flocculent aggregates rather than compact clumps (Fig. 6b). These observations suggest that the altered sedimentation phenotype of the CB OE mutant may result from its distinct S-layer or increased exopolysaccharide production.

Collectively, the remarkable growth enhancement of the CB OE strain under high CO_2_ and nutrient-rich (5× BG) conditions suggests its potential applications in the metabolic engineering of cyanobacteria for improved biomass production and CO_2_ utilization. Notably, these conditions diverge from natural or industrial cultivation environments, highlighting the need for further optimization and in-depth evaluation in a more physiologically relevant context.

## Conclusion

Rubisco catalyzes the first step of CO_2_ assimilation in the Calvin–Benson cycle and is the primary biochemical limitation of photosynthetic carbon fixation. Previous efforts to increase Rubisco content or the growth of cyanobacteria have often met limited success (Liang and Lindblad 2016, 2017). In this study, we demonstrate that targeted upregulation of β-carboxysome proteins in Syn6803, together with altered stoichiometries of CcmM73/CcmM48 and other structural components, substantially altered in vivo carboxysome morphology, photosynthetic efficiency, and cell growth under elevated CO_2_ and nutrient-enriched conditions, leading to profound molecular and physiological remodeling.

Despite our findings that high CO_2_ and nutrient-rich conditions improved photosynthesis and cell growth of the CB OE and shell OE mutants, the precise regulation of in vivo protein expression and the mechanistic links between specific protein abundance changes and observed phenotypic improvements merit comprehensive investigation. Moreover, the roles of environmental factors in modulating intracellular β-carboxysome proteins merit further elucidation. The advanced knowledge will facilitate rational design and development of fast-growing cyanobacterial chassis cells under various environmental conditions.

In summary, our study provides insights into how engineered carboxysome biogenesis is integrated with broader cellular networks that control energy metabolism and protein homeostasis. These findings establish a rational framework for tuning carboxysome assembly using synthetic biology to enhance carbon fixation and cyanobacterial growth under optimized conditions and will aid in the engineering of functional carboxysomes and carboxysome-based CCM into heterologous hosts offers a promising strategy to boost photosynthetic efficiency and crop productivity (Hanson et al. 2016; Chen et al. 2023a; Nguyen et al. 2024; Nguyen et al. 2025a).

## Materials and methods

### Bacterial strains, growth conditions, and physiology

The cyanobacterium Syn6803 cultures were grown at 30 °C with constant illumination of a light intensity of 40 *μ*mol photons m^−2^ s^−1^ in BG11 medium in culture flasks with constant shaking at 120 rpm. Solid BG11 medium was prepared by adding 1.5 (w/v) Bacto-Agar (BD Diagnostics). Cultures were grown in air without an additional CO_2_ source, except for the growth assays under CO_2_ treatment, in which Syn6803 cultures were cultivated in the incubators aerated with 2% (v/v) CO_2_.

The cell growth curve was performed with a starting OD_730_ of 0.1 in 30 mL cultures using regular BG11 and 5× BG media. Where appropriate, apramycin (Apra), kanamycin (Kan), or spectinomycin (Spec) was added to the medium at a final concentration of 25 *μ*g mL^−1^. Chlorophyll fluorescence kinetics were measured on an AquaPen-C fluorometer (Photon Systems Instruments) using cells diluted to a chlorophyll *a* concentration of 1 to 2 *μ*g mL^−1^ and dark-adapted for 10 min. The rate of photosynthetic O_2_ evolution was measured using an OxyLab system (Hansatech Instruments Ltd., Norfolk, UK). All data are reported in *μ*mol O_2_ min^−1^ OD^−1^.

One milliliter of the samples was centrifuged at 10,000 × *g* for 5 min. The pellets were resuspended in an equal volume of absolute methanol and homogenized by vortexing and incubated in the dark at 4 °C for 24 h. After incubation, the cell solutions were centrifuged at 12,000 × *g* for 5 min at 4 °C. The absorbance of the supernatant was measured by Cary 3500 Compact UV–Vis spectrophotometer (Agilent Technologies, CA, USA). The chlorophyll *a* and carotenoid (Car) concentrations were calculated using Eqs. 1 and 2 (Lichtenthaler 1987):


(1) $$\mathrm{chlorophyll}\;a(\mu g\;mL^{-1})=16.72OD_{665}\text{-}9.16OD_{652}$$

(2) $$\mathrm{Car}\;(\mu g\;mL^{\text{-}1})=(1000OD_{470}\text{-}2.27\;\mathrm{chlorophyll}\;a\text{-}81.4\;(18.61OD_{645}\text{-}3.96OD_{662}))/227$$

### Cloning of constructs

All fragments needed for cloning the vectors were amplified using CloneAmp HiFi PCR Premix (TAKARA) according to the manufacturer's protocol. All the primers and templates used for PCR amplification are listed in Table S1. Fragments were then ligated using NEBuilder HiFi DNA Assembly (New England Biolabs) according to the manufacturer's protocol and transformed into competent *Escherichia coli* (*E. coli*)cells, grown in LB medium at 37 °C, and supplemented with appropriate antibiotics (50 *μ*g mL^−1^ Kan or 100 *μ*g mL^−1^ Amp). The 3,062 bp fragments of the *slr0168* gene and its upstream and downstream sequences were amplified from the Syn6803 genome and subcloned into the pGEM vector using *slr0168*-pGEM-F/R and ampR-ori-F/R primers to obtain the pGEM-slr0168 plasmid. Then, the fragments of the *psbA2* promoter, the *rbcL* terminator, and the kanamycin gene produced in a 2-step PCR process using primers PpsbA2-KmR-F/PpsbA2-TrbcL-R, TrbcL-PpsbA2-F/TrbcL-0168-R, and KmR-0168-F/KmR-PpsbA2-R were ligated into the *EcoRI* site of *slr0168* to obtain the pGEM-slr0168-KmR-PpsbA2-TrbcL plasmid. Then, the fragments of *rbcLXS-ccaA*, *ccmKLMN*, and *ccmK-O-P-S* were produced in a 2-step PCR process using *rbcLXS*-F/R, *ccaA*-F/R, *ccmKLMN*-F/R, *ccmK3K4*-F/R, *ccmO*-F/R, *ccmP*-F/R, and *ccmS*-F/R primers, followed by insertion into the *EcoRV* site of the pGEM-slr0168-KmR-PpsbA2-TrbcL plasmid to obtain the CB OE plasmid used for overexpression of carboxysomes. The shell OE plasmid was constructed by fusing the fragments of *ccmKLMN* and *ccmK*-O-P-S. The CB OE and shell OE plasmids were transferred to WT cells by homologous recombination, and transformants were selected on BG11 agar plates containing 25 *µ*g ml^−1^ Kan.

Segregation was evaluated by PCR using the primers seq-slr0168-2/seq-Trbcl-P*psbA2*-R, seq-*rbcL*-F/*ccaA*-R, seq-carbox-5/seq-*ccmM*-R, seq-trc-F1/seq-*ccmM*-R, seq-carbox-8/*ccmK3K4*-R, and seq-carbox-12/seq-Trbcl-P*psbA2*-R. All the primers used in this analysis are listed in Table S2.

### Carbohydrate content measurement

Total carbohydrate content was determined spectrophotometrically by the phenol-sulfuric acid method using a glucose-based standard curve as described previously (Hu et al. 2023b). Briefly, 30 mL of cyanobacterial cultures were pelleted by centrifugation at 6,000 × *g* for 10 min and lyophilized with Heto PowerDry LL3000 freeze-dryer (Thermo, Denmark) for 24 h. Then, 10 mg frozen sample was weighed and resuspended in 40 mL deionized water to prepare a 0.25 mg mL^−1^ suspension. A 1 mL of suspension was mixed with 5 mL sulfuric acid (96% v/v) and 1 mL phenol solution (5% w/v) and then incubated in a water bath at 90 °C for 5 min. Absorbance was read at 490 nm in a FLUOstar Omega microplate reader (BMG Labtech, Offenburg, Germany).

### Protein extraction and immunoblot analysis

Cyanobacterial cultures were harvested by centrifugation (6,000 × *g* for 10 min) and resuspended in 40 mM Tris-HCl buffer (pH 8.0). The cells were then broken by oscillation with glass beads (Sigma, 150 to 212 *μ*m) at 4 °C for 20 min (1 min on + 1 min off for 10 cycles). The supernatant was centrifuged (12,000 × *g* for 10 min at 4 °C) to separate the soluble fraction from the pellet. Protein concentrations of the soluble fraction were determined using the DS-11 spectrophotometer (DeNovix, Wilmington, USA), and 40 *μ*g of soluble protein was separated on 15% SDS-PAGE. Gels were blotted onto Amersham Hybond PVDF membrane (GE Healthcare) at 95 V for 60 min. The membrane was immunoprobed using rabbit polyclonal antisera against RbcL (1:5,000; Agrisera AS03 037), CcmK2 (1:5,000; -PhytoAB PHY5336S), or CcmM (1:5,000; PhytoAB PHY5331S) overnight, followed by goat anti-rabbit HRP-conjugated secondary antibody (1:5,000; Agrisera AS09 602) for 1 h. Immunoreactive polypeptides were visualized using Clarity Western ECL substrate (Bio-Rad), and signals were captured with an ImageQuant LAS4000 biomolecular imager (GE Healthcare). Signal quantification was performed using Fiji ImageJ.

### Electron microscopy

For TEM, 30 mL of cyanobacterial cell cultures were pelleted by centrifugation (6,000 × *g* for 10 min) and washed by resuspending the pellet in 10 mL 0.1 M sodium cacodylate buffer at pH 7.2 twice. The cells were first fixed with 2.5% glutaraldehyde in 0.1 M sodium cacodylate buffer overnight. After agarose embedding, the samples were stained with 2% osmium tetroxide and 3% potassium ferrocyanide using 3 steps for 1 h. The osmium stain was set using 1% thiocarbohydrazide and 2% osmium tetroxide. The samples were stained with 2% uranyl acetate before dehydration by increasing the ethanol concentration from 30% to 100% and increasing resin embedding. Thin (70 nm) sections were cut with a diamond knife and poststained with 3% lead citrate. For isolated carboxysomes, samples were immobilized onto the glow-discharged grids and then stained with 2% uranyl acetate. TEM imaging was conducted using an FEI Tecnai G2 Spirit BioTWIN transmission electron microscope equipped with a Gatan Rio 16 camera. The diameters of carboxysomes were measured using Fiji ImageJ.

For SEM, 30 mL of cyanobacterial cell cultures were pelleted by centrifugation (6,000 × *g* for 10 min) and washed by resuspending the pellet in 40 mM Tris-HCl buffer twice. The cells were first fixed with 2.5% glutaraldehyde in 0.1 M sodium cacodylate buffer at 4 °C for 1 h. Then the samples were dehydrated by increasing the ethanol concentration from 50% to 100%, followed by resuspending the pellet with 200 *μ*L of 100% ethanol. To prepare a specimen for SEM characterization, 10 *μ*L of the mixture was pipetted on top of a silica disc. Then the dried specimen was coated with chromium for 15 s by Quorum Q150T ES spray device (Quorum, Laughton, UK). SEM measurements were performed on a Hitachi S4800 cold field emission (FE) scanning electron microscope, and imaging was conducted at a working voltage of 3.0 kV and a working distance of 8 mm using a combination of upper and lower secondary electron detectors.

### Live-cell fluorescence microscopy and image analysis

Fluorescence microscopy was performed on a ZEISS Elyra 7 with Lattice SIM at 488 nm for GFP using 5 *μ*L of cells spotted onto 1% agarose pads. Live-cell images were recorded from at least 3 different cultures. All images were captured with all pixels below saturation. Automated analysis of the carboxysome number and RbcL fluorescence in vivo was carried out using Fiji ImageJ with a custom-programmed plugin. More than 230 cells were analyzed from 3 different images.

### Statistical analysis

Data were processed in Prism v8.0 (GraphPad Software, La Jolla, CA, USA) and are presented as means ± Sd or means of 3 replicates. Statistical differences between WT and mutants were determined by 1-way ANOVA followed by Dunnett's test (*P < 0.05, **P < 0.01, ***P < 0.001, and ****P < 0.0001).

## Supplementary Material

kiag261_Supplementary_Data

## Data Availability

The data supporting the findings of this study are available within the article and/or its supplementary materials.
