# Supplementary material for: Overexpression of β-carboxysomes increases photosynthesis and growth in Synechocystis sp. PCC 6803
Source: Plant Physiol. 2026 May 6;201(3):kiag261. doi: 10.1093/plphys/kiag261 (PMC13332109; doi:10.1093/plphys/kiag261)
Supplement: kiag261_Supplementary_Data [file kiag261_supplementary_data.pdf]

Supplementary Data

Jinlu Hu[1,2], Kuo Zhao[2], Yu Chen[2], Xingwu Ge[2], Jing Yang[2,3], Gregory F. Dykes[2], Jian-Yu Shi[1], Lu-Ning Liu[2,4]*

[1]School of Life Science and Technology, Northwestern Polytechnical University, Xi'an, Shaanxi 710072, China

[2]Institute of Systems, Molecular and Integrative Biology, University of Liverpool, Liverpool L69 7ZB, United Kingdom

[3]Materials Innovation Factory and Department of Chemistry, University of Liverpool, Liverpool L7 3NY, United Kingdom

[4]MOE Key Laboratory of Evolution and Marine Biodiversity, State Key Laboratory of Marine Food Processing and Safety Control & College of Marine Life Sciences, Ocean University of China, Qingdao, China

*Corresponding author: luning.liu@liverpool.ac.uk

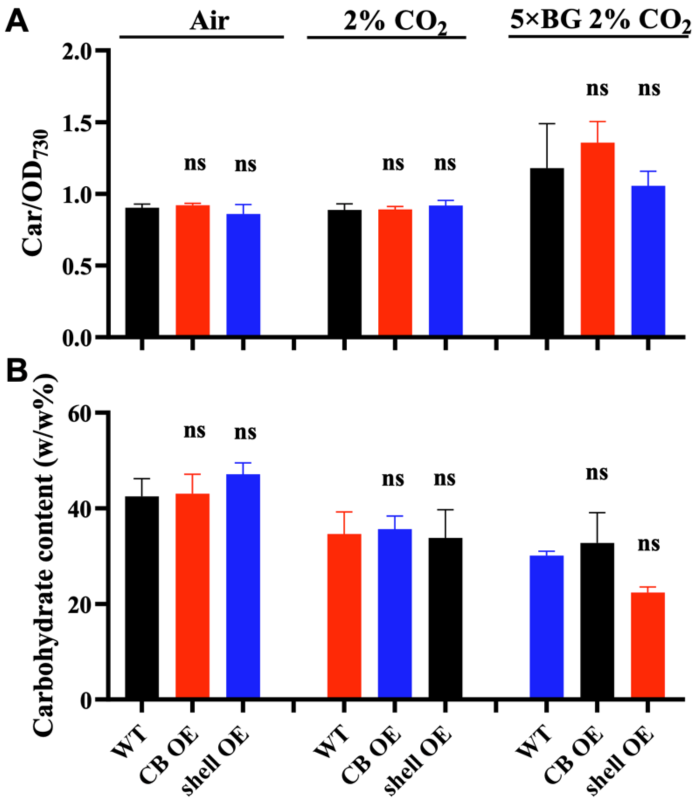

Supplementary Figure S1. Comparison of carotenoids and carbohydrate content of CB OE and shell OE mutants under air or presence of 2% CO_2. A. Carotenoids. B. Carbohydrate. 5×BG 2% CO_2: Strains were grown under 5×BG and 2% CO_2 conditions. Data points with error bars represent mean of n=3 biological replicates ± SD. ns = no significance, $p > 0.05$.

Supplementary Table S1. Oligonucleotides used in this study.

Name	Sequence (5'→3')	Purpose
ampR-ori-F	gcaggaaagaacatgtgagc	Constructing pGEM-slr0168 plasmid
ampR-ori-R	gtatttcacaccgcatcagg	Constructing pGEM-slr0168 plasmid
slr0168/pGEM-F	cctgatgcggtgtgaaatactcaccaaaatcagttccagcaca	Constructing pGEM-slr0168 plasmid
slr0168/pGEM-R	gctcacatgttctttcctgctgctctggtttctaggtcttctg	Constructing pGEM-slr0168 plasmid
KmR-0168-F	ttgatggttatatgggaattcctccttagttcctattccgaagt	Constructing pGEM-slr0168-KmR-PpsbA2-TrbcL plasmid
KmR-PpsbA2-R	atacctaatacttattgatagtgtaggctggagctgc	Constructing pGEM-slr0168-KmR-PpsbA2-TrbcL plasmid
PpsbA2-KmR-F	gaagcagctccagcctacactatcaataagtattaggtatatggatcata	Constructing pGEM-slr0168-KmR-PpsbA2-TrbcL plasmid
PpsbA2-TrbcL-R	ccgacaatccaaacaccggtgatatcttggttataattccttatgtatttgt	Constructing pGEM-slr0168-KmR-PpsbA2-TrbcL plasmid
TrbcL-PpsbA2-F	acataaggaattataaccaagatatcaccggtgtttggattgtc	Constructing pGEM-slr0168-KmR-PpsbA2-TrbcL plasmid
TrbcL-0168-R	tgccatccataccgggaattgctgtcgaagttgaacatc	Constructing pGEM-slr0168-KmR-PpsbA2-TrbcL plasmid
rbcLXS-F	taaggaattataaccaagatctattcaatgtttatggaggactgac	Constructing CB OE plasmid
rbcLXS-R	ttccagccttcttagtaacggccttggttttg	Constructing CB OE plasmid
ccaA-F	ccgttactaagaaggctggaattccatcaac	Constructing CB OE plasmid
ccaA-R	ctcctagaatcgtgaaagtgctaacgggagcctcgataa	Constructing CB OE plasmid
ccmKLMN-F	tttatcgaggctcccgttagcactttcacgattctaggagac	Constructing CB OE plasmid
ccmKLMN-R	tctagggaaacggatggaaattaagaactaaaatatcgtcgttccg	Constructing CB OE or shell OE plasmid
ccmK34-F	gacgatattttagttcttaatttccatccgtttccctagag	Constructing CB OE or shell OE plasmid
ccmK34-R	tctatgataacccattaacgacgatttaaaccttctg	Constructing CB OE or shell OE plasmid
ccmO-F	aaatcgtcgttaatgggttatcatagaaggcga	Constructing CB OE or shell OE plasmid

ccmO-R	gcaaaactatggcctaatcaatttctggacctagcac	Constructing CB OE or shell OE plasmid
ccmP-F	agaaattgattaggccatagttttgcatttccac	Constructing CB OE or shell OE plasmid
ccmP-R	aacttacttccgagcctccattattcatttttac	Constructing CB OE or shell OE plasmid
ccmS-F	tggaggctcggaagtaagttcacccccc	Constructing CB OE or shell OE plasmid
ccmS-R	acaatccaaacaccggtgatatcctaggctagtttttcctggag	Constructing CB OE or shell OE plasmid
ccmKLMN-PpsbA-F	taaggaattataaccaagatcactttcacgattctaggagac	Constructing shell OE plasmid

Supplementary Table S2. Primers for PCR verification used in Figure 1. F, forward; R, reverse.

**Name**	**Sequence (5'→3')**	**Purpose**
seq-slr0168-2	gatccatgacattgacctgttg	F primer of lanes 1, 2, 3
seq-Trbcl-PpsbA2-R	gccacattgttgtcaaaggc	R primer of lanes 1, 2, 3
seq-rbcL-F	gcgaactctggaaagagatc	F primer of lane 4
ccaA-R	ctcctagaatcgtgaaagtgctaacgggagcctcgataa	R primer of lane 4
seq-carbox-5	aactcaactctctccaggaa	F primer of lane 5
seq-trc-F1	tacgtgttccgcttcctttag	F primer of lane 8
seq-ccmM-R	caccgtggcaataacctgg	R primer of lanes 5, 8
seq-carbox-8	aaccaaatcttggcagagc	F primer of lanes 6, 9
ccmK34-R	tctatgataacccattaacgacgatttaaaccttctg	R primer of lanes 6, 9
seq-carbox-12	ttattggagatggcctttgcac	F primer of lanes 7, 10
seq-Trbcl-PpsbA2-R	gccacattgttgtcaaaggc	R primer of lanes 7, 10
